# Patient-reported utilities in advanced or metastatic melanoma, including analysis of utilities by time to death

**DOI:** 10.1186/s12955-014-0140-1

**Published:** 2014-09-10

**Authors:** Anthony J Hatswell, Becky Pennington, Louisa Pericleous, Donna Rowen, Maximilian Lebmeier, Dawn Lee

**Affiliations:** BresMed, Sheffield, UK; Bristol-Myers Squibb Pharmaceuticals Limited, Uxbridge, UK; School of Health and Related Research (ScHARR), University of Sheffield, 84 Queen Street, Sheffield, S1 2DW UK

**Keywords:** Preference-based utilities, EORTC-8D, SF-6D, Melanoma, Ipilimumab, MDX010-20

## Abstract

**Background:**

Health-related quality of life is often collected in clinical studies, and forms a cornerstone of economic evaluation. This study had two objectives, firstly to report and compare pre- and post-progression health state utilities in advanced melanoma when valued by different methods and secondly to explore the validity of progression-based health state utility modelling compared to modelling based upon time to death.

**Methods:**

Utilities were generated from the ipilimumab MDX010-20 trial (Clinicaltrials.gov Identifier: NCT00094653) using the condition-specific EORTC QLQ-C30 (via the EORTC-8D) and generic SF-36v2 (via the SF-6D) preference-based measures. Analyses by progression status and time to death were conducted on the patient-level data from the MDX010-20 trial using generalised estimating equations fitted in Stata®, and the predictive abilities of the two approaches compared.

**Results:**

Mean utility showed a decrease on disease progression in both the EORTC-8D (0.813 to 0.776) and the SF-6D (0.648 to 0.626). Whilst higher utilities were obtained using the EORTC-8D, the relative decrease in utility on progression was similar between measures. When analysed by time to death, both EORTC-8D and SF-6D showed a large decrease in utility in the 180 days prior to death (from 0.831 to 0.653 and from 0.667 to 0.544, respectively). Compared to progression status alone, the use of time to death gave similar or better estimates of the original data when used to predict patient utility in the MDX010-20 study. Including both progression status and time to death further improved model fit. Utilities seen in MDX010-20 were also broadly comparable with those seen in the literature.

**Conclusions:**

Patient-level utility data should be analysed prior to constructing economic models, as analysis solely by progression status may not capture all predictive factors of patient utility and time to death may, as death approaches, be as or more important. Additionally this study adds to the body of evidence showing that different scales lead to different health state values. Further research is needed on how different utility instruments (the SF-6D, EORTC-8D and EQ-5D) relate to each other in different disease areas.

**Electronic supplementary material:**

The online version of this article (doi:10.1186/s12955-014-0140-1) contains supplementary material, which is available to authorized users.

## Background

Utilities are commonly used to provide a preference weighted estimate of health-related quality of life (HRQL) benefits provided by treatments in the context of assessing the cost effectiveness of those treatments. They are therefore crucial to health technology assessment when using a cost per quality-adjusted life year framework. Utility values can be generated in several ways, including generic preference-based measures, condition-specific preference-based measures, vignettes and directly-elicited patient utility values. However, the use of different instruments (in the same population) has been shown to produce different results, with a range of utility measures having been used in oncology [1-3].

Health-state classifications for economic modelling are frequently based on a patient’s progression status, often the primary endpoint in oncology trials (including melanoma). Melanoma is a form of skin cancer and if identified early the prognosis is generally good [4]; however, for advanced melanoma survival is poor and options for treatment are limited. In oncology progression is generally accepted to be important for both disease burden and HRQL, with a delay in progression an objective of treatment. During our investigation we sought clinical opinion, which indicated that there is a decline in HRQL in the final months of life of advanced melanoma patients, which may not be appropriately captured solely through the use of progression-based heath states.

We therefore undertook the study reported here with two objectives in mind: firstly to report and compare pre- and post-progression health state utilities in advanced melanoma, when generated using different methods - via the EORTC (European Organisation for Research and Treatment of Cancer, Brussels, Belgium), and the SF-36 (QualityMetric, Lincoln, USA); secondly, to explore the validity, in advanced melanoma, of progression-based health-state utility modelling compared to modelling based upon time to death health states.

## Methods

Regulatory approval for ipilimumab in advanced (unresectable or metastatic) melanoma was based on data from the Phase III clinical trial MDX010-20 (Clinicaltrials.gov Identifier: NCT00094653), conducted in accordance with the Declaration of Helsinki, and the laws and regulatory requirements of the countries where the research was performed. Patients (or their legal representatives) were required to give written informed consent before enrolment, and the protocol approved by institutional review boards at each of the 125 sites involved. All patients enrolled in to the study had unresectable advanced melanoma (disease that had spread from the area of skin where it originated), at stage III (the disease having spread to regional lymph nodes, but no evidence of distant metastases), or stage IV (the disease having spread to distant lymph nodes, or other organs).

Trial data were extracted from the study database using SAS® (SAS, Cary, USA) and analysed in Stata® 12.0 (StataCorp, Texas, USA) [5]. The trial included 676 patients, randomised 3:1:1 to ipilimumab + gp100, ipilimumab only, or gp100 only. Gp100 is an experimental peptide-based vaccine, with few side effects, later shown to be ineffective in increasing overall survival. Approximately 60% of patients were male, with the majority of patients having Eastern Cooperative Oncology Group (ECOG) performance status 0 (55%) or 1 (43%). All patients had received prior systemic therapy for their advanced melanoma – a complete description of the study is available in Hodi et al., 2010 [5]. In the study, patients were asked to complete both the EORTC QLQ-C30 and SF-36v2 questionnaires on receipt of the first dose of treatment, at the end of treatment and 12 weeks after treatment. These time points were pre-specified for the first 4 doses, however beyond this point quality of life instruments were only administered when patients received retreatment.

The EORTC QLQ-C30, administered in MDX010-20, is frequently used in oncology clinical trials as it captures many of the symptoms commonly seen in cancer patients, such as nausea, pain and fatigue, as well as generic aspects of function (including physical, emotional and role). Rowen et al. generated the EORTC-8D – a condition-specific preference-based measure derived from the EORTC QLQ-C30 [6]. A sample of health states were valued by 350 members of the UK general public using time trade-off, and these values were modelled to produce utilities for all health states. In order to generate EORTC-8D utilities from the EORTC QLQ-C30 results, Model 3 from the mapping by Rowen et al. was used [6]. In the MDX010-20 trial, this questionnaire was completed by 616 patients (1,237 observations).

The SF-6D (derived from the SF-36 or SF-12) is a widely-used generic preference-based measure of HRQL, which has been valued by 611 members of the UK general public using standard-gamble methodology for a sample of health states. Several methods are available to convert SF-36 results to SF-6D utilities; this study uses the most recent non-parametric Bayesian method [7-9]. In the MDX010-20 trial, 599 patients completed the SF-36 (1,205 observations).

The majority of HRQL questionnaires (both EORTC QLQ-C30 and SF-36) were completed within the first 24 weeks of the trial (before and after patients were administered their initial course of ipilimumab), though observations are available for up to 4 years (5% of patients completed questionnaires after 12 months if receiving retreatment). Where multiple observations were available for a patient, all observations were included in calculations. The level of non-response to the EORTC QLQ-C30 and SF-36 was low within the study, at 7.8% and 11.4%, respectively. We did not attempt to impute missing values, as their low number means they would be unlikely to affect the results of the study, nevertheless this remains a limitation of the work performed.

To provide context to the analysis, a literature search was completed in May 2013 for utilities in advanced melanoma (including previously untreated disease) with at least 30 patients in the study. This search included the Medline, Embase, NHS Health Economic Evaluation Database, Cochrane Health Technology Assessment, Database of Abstracts of Reviews of Effect (DARE), Cumulative Index to Nursing and Allied Health Literature (CINAHL) and Econlit databases.

### Progression status versus time to death

In order to ascertain whether the time to a patient’s death influenced their quality of life, a series of regression models were fitted in Stata. Patients were split into six groups based on their time to death after the HRQL measurement was taken. The groups were based on clinician feedback on the phases of life generally seen in patients before death (over 180 days, 120–180 days, 90–120 days, 60–90 days, 30–60 days, and under 30 days). Patient time to death was then extracted from the study database and grouped according to these time points.

Where a patient was still alive at the end of the study, the final measurement point was used as the time of death. In 91% of cases where censoring occurred, this put patients in the health state furthest from death (>180 days), with only 2.9% of EORTC-8D and 2.5% of SF-6D of observations categorised as censored before the 180 days from death time point. Given this low level of censoring models accounting for censoring were not required to be fitted.

The models fitted to the data were generalised estimating equation (GEE) random effects models to account for correlation between repeated measurements from the same individuals. GEE models were chosen instead of generalised linear mixed models, in order to produce population averages as required for the purpose of health technology assessment and economic modelling. To compare model fit the mean absolute error and the root mean squared error were calculated, with tests for significance using Student’s *t*-test with a p-value of 0.05.

## Results

### EORTC-8D versus SF-6D values

**Figure 1:**
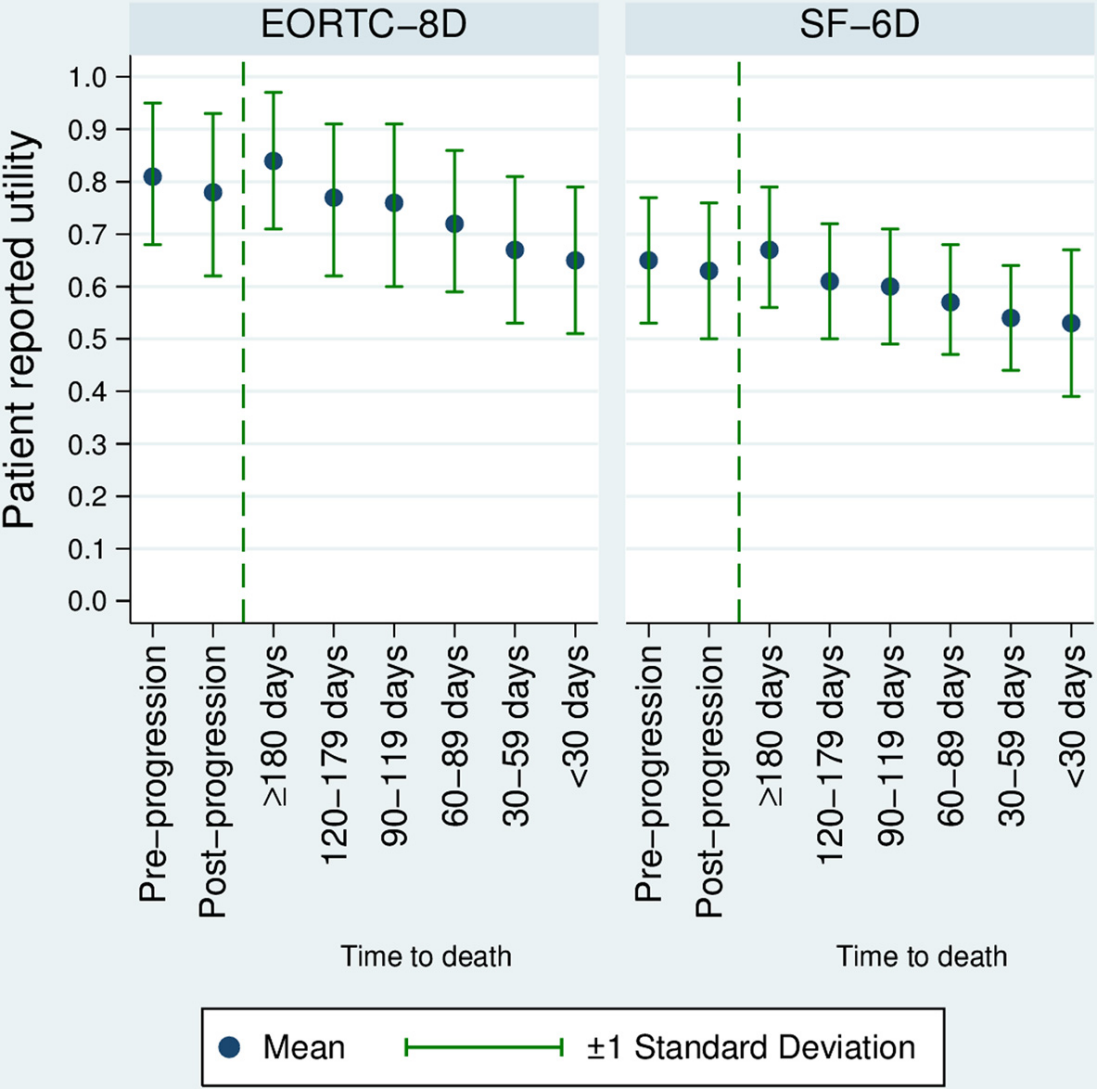
**Plot of EORTC-8D and SF-6D patient reported utilities by progression status and time to death in the MDX010-20 trial.**

**Figure 2:**
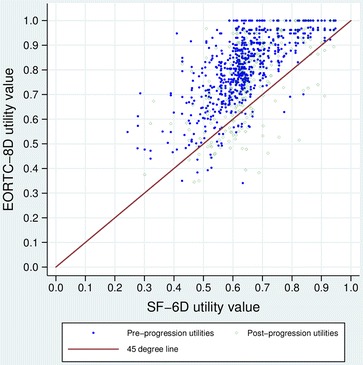
**Plot of EORTC-8D utility versus SF-6D utility when measured at identical time points, by progression status.**

In all treatment groups and disease states, mean utilities generated from the EORTC-8D were significantly higher than those seen with the SF-6D (p < 0.001), the difference in the values being shown in Figure 1. Of 807 measurements of HRQL at the same time-point for both instruments, the EORTC-8D utility was higher on 777 occasions (Figure 2). SF-6D values appear to be more concentrated around the mean: 28% of the SF-6D utilities were within 5% of the mean value, compared to only 13% of the EORTC-8D utilities, though no statistical tests were performed to estimate this formally. Despite the differences in magnitude between the two methods, the change in utility on disease progression was similar with both the EORTC-8D utilities (−4.6%) and SF-6D utilities (−3.3%).

### Progression status versus time to death

Three models were fitted to the patient-level data for both EORTC-8D and SF-6D in order to predict patient utility, these are shown in Table 1. Model 1 used progression status alone to predict utility, Model 2 used time to death health states only, and Model 3 used both progression status and time to death health states.

**Table 1:** **Results of regression analyses based for models, including goodness-of-fit statistics**

**EORTC-8D derived results**						
	**Progression (model 1)**		**Time to death (model 2)**		**Progression + time to death (model 3)**	
Constant	0.803		0.831		0.837	
(SE)	(0.006)		(0.006)		(0.006)	
Progression	−0.048				−0.029	
(SE)	(0.007)				(0.007)	
120 - 179 days to death			−0.060		−0.059	
(SE)			(0.012)		(0.012)	
90 - 119 days to death			−0.068		−0.065	
(SE)			(0.015)		(0.015)	
60 - 89 days to death			−0.112		−0.106	
(SE)			(0.016)		(0.016)	
30 - 59 days to death			−0.153		−0.142	
(SE)			(0.015)		(0.015)	
Under 30 days to death			−0.178		−0.165	
(SE)			(0.019)		(0.019)	
	MAE	RMSE	MAE	RMSE	MAE	RMSE
All values	0.119	0.145	0.108	0.132	0.108	0.132
0.9 < EORTC-8D ≤ 1 (n = 354)	0.167	0.171	0.137	0.145	0.139	0.147
0.8 < EORTC-8D ≤ 0.9 (n = 280)	0.056	0.066	0.046	0.063	0.048	0.064
0.65 < EORTC-8D ≤ 0.8 (n = 332)	0.061	0.073	0.077	0.088	0.074	0.086
0.0 < EORTC-8D ≤ 0.65 (n = 174)	0.235	0.246	0.206	0.225	0.204	0.222
**SF-6D derived results**						
	Progression (model 1)		Time to death (model 2)		Progression + time to death (model 3)	
Constant	0.642		0.667		0.670	
(SE)	(0.006)		(0.005)		(0.005)	
Progression	−0.030				−0.016	
(SE)	(0.005)				(0.006)	
120 - 179 days to death			−0.050		−0.050	
(SE)			(0.010)		(0.010)	
90 - 119 days to death			−0.059		−0.057	
(SE)			(0.013)		(0.013)	
60 - 89 days to death			−0.089		−0.085	
(SE)			(0.014)		(0.013)	
30 - 59 days to death			−0.119		−0.113	
(SE)			(0.013)		(0.013)	
Under 30 days to death			−0.134		−0.126	
(SE)			(0.016)		(0.016)	
	MAE	RMSE	MAE	RMSE	MAE	RMSE
All values	0.091	0.124	0.102	0.134	0.101	0.133
0.75 < SF-6D ≤ 1 (n = 201)	0.201	0.210	0.199	0.212	0.199	0.212
0.65 < SF-6D ≤ 0.75 (n = 228)	0.050	0.057	0.047	0.065	0.047	0.065
0.55 < SF-6D ≤ 0.65 (n = 435)	0.028	0.037	0.051	0.058	0.049	0.057
0 < SF-6D ≤ 0.55 (n = 208)	0.159	0.174	0.175	0.194	0.173	0.192

SE = standard error, MAE = mean absolute error, RMSE = root mean squared error.

Model 1 showed that patients experienced a reduction in utility of 0.048 (standard error 0.007) following progression using the EORTC-8D and a reduction in utility of 0.030 (standard error 0.005) using the SF-6D. Clinician feedback following discussion of these results was that patients would be treated according to their expected survival period. These health states were specified by clinicians and then used in Model 2 and Model 3, as described in the Methods section.

Model 2 used time to death as a categorical variable, grouping patients by survival period, and showed that patient utility fell consistently as a patient approached death in both EORTC-8D and SF-6D utilities. This slightly improved the predictive power of the regression in the EORTC-8D, and slightly worsened it in the SF-6D. Model 3 included whether a patient had progressed as a dummy variable, and their time to death at the point the measurement was taken, and improved on the time to death model for both EORTC-8D and SF-6D. The results of this time to death including progression model showed a similar pattern of decline in utility as a patient approaches death, with an additional decrement to utility if a patient had experienced disease progression. The fit of each of the models can be seen in Table 2, which compares predicted values for each of the health state based models, to observed values in those patient groups. Model fit can also been seen in Table 1, where the mean absolute error and root mean squared error are given for all values, and also reported separately for approximately equal segments of the utility distributions, which allows a comparison of model fit across the distribution of utility values.

**Table 2:** **Predicted versus observed mean utility values for models based on health states**

**Model**	**Health state**	**EORTC-8D**		**SF-6D**	
		**Predicted**	**Observed**	**Predicted**	**Observed**
Model 1	Pre-progression	0.803	0.813	0.642	0.648
	Post-progression	0.755	0.776	0.612	0.626
Model 2	180 or more days to death	0.831	0.840	0.667	0.672
	120 - 179 days to death	0.771	0.767	0.616	0.610
	90 - 119 days to death	0.763	0.756	0.613	0.600
	60 - 89 days to death	0.720	0.723	0.585	0.574
	30 - 59 days to death	0.679	0.670	0.557	0.541
	Under 30 days to death	0.653	0.651	0.544	0.531
Model 3	*Pre-progression*				
	180 or more days to death	0.837	0.848	0.670	0.677
	120 - 179 days to death	0.779	0.777	0.620	0.615
	90 - 119 days to death	0.772	0.759	0.613	0.591
	60 - 89 days to death	0.731	0.737	0.585	0.588
	30 - 59 days to death	0.695	0.690	0.557	0.554
	Under 30 days to death	0.672	0.629	0.544	0.518
	*Post-progression*				
	180 or more days to death	0.808	0.820	0.654	0.661
	120 - 179 days to death	0.749	0.742	0.614	0.595
	90 - 119 days to death	0.743	0.750	0.613	0.623
	60 - 89 days to death	0.702	0.693	0.585	0.547
	30 - 59 days to death	0.665	0.643	0.507	0.521
	Under 30 days to death	0.642	0.675	0.534	0.547

Models including death as a continuous variable, or restricting analysis only to patients who died in the study period did not produce substantially different results (results not presented). Adding further clinically-relevant variables (treatment assignment, prior Interleukin-2 usage, age, ECOG status or melanoma stage: all measured at baseline) did not result in significant coefficients or increase the predictive power of the model. These additional variables were therefore not included in the final models, but are provided in Additional file 1: Table S1 for completeness. Fitting models separately for pre- and post-progression utilities when including time to death yielded similar coefficients for pre-progression and post-progression - the consistency of parameter estimates and standard errors across models when including progression status (moving from Model 2 to Model 3) indicate low levels of multi-collinearity between progression status and time to death.

Utility remained high until approximately 180 days before death with both the EORTC-8D and SF-6D (0.840 and 0.672), similar to values seen in the general population (0.80 for a 55–64 year old using the EQ-5D) [10]. From this point onwards HRQL continually decreased, with the final 30 days of life having the largest decrease compared to patients 180 days or more from death (EORTC-8D −0.189 and SF-6D −0.141) (Table 1). All time based health state coefficients were significantly lower than the 180 days before death health state (p < 0.001 for all coefficients), for both EORTC-8D and SF-6D.

For the EORTC-8D, the time to death health state approach provides a more accurate estimate of patient utility than progression status according to the mean absolute error and root mean squared error (particularly for values further from the mean). Using both factors further increases accuracy of estimates. For the SF-6D, using time to death as health states resulted in predictions on average slightly worse than the progression based model. Using both time to death and progression improved the accuracy of estimates compared to time to death alone, though did not provide as good a prediction as progression only.

## Discussion

### EORTC-8D versus SF-6D utility values

No studies are presently available that directly compare the EORTC-8D to the SF-6D. We observed that EORTC-8D utilities were higher than SF-6D utilities in both the pre- and post-progression states. This difference in utilities derived from the two preference-based measures adds to the body of literature that suggests different measures may produce different utilities. EQ-5D, which like EORTC-8D is valued using time trade-off, has been shown to produce different utilities to SF-6D (both higher and lower) in rheumatoid arthritis, constipation and herniated discs [1-3,11].

A potential explanation for the difference in utilities generated by the EORTC-8D is the classification system used - this was originally derived using a multiple-myeloma dataset [6]. When compared to the original data, patients within the MDX010-20 dataset were more likely to respond that they have the best level of health, and less likely to report that they have the lowest level of health for the physical, role functioning, and pain dimensions, than those from the EORTC-8D source dataset. Although this does not in itself mean that the classification system is not appropriate, further research is encouraged to investigate whether the EORTC-8D has desirable psychometric properties when applied to other cancers.

Investigation of the properties of EORTC-8D- is particularly important as the EORTC QLQ-C30 is a frequently-used measure in oncology clinical trials, therefore utilities from the EORTC-8D will often be available, whereas a generic instrument (such as the SF-36 or EQ-5D) may not be included in a study. Equally, for health technology assessment purposes, it is likely to be important to understand how the EORTC-8D relates to the SF-6D, Health Utilities Index 3 (HUI-3) and, most notably, the EQ-5D (the preferred measure of the National Institute of Health and Care Excellence [NICE], and most frequently used utility measure in health technology assessment studies internationally) [12-14]. At present two studies are available comparing the EORTC-8D and EQ-5D, though only in two types of cancer [13,15], however there is the potential for further analyses from a recently completed study of dabrafenib compared to dacarbazine in advanced melanoma, the BREAK-3 study, as this included both EORTC-8D and EQ-5D [16].

### Patient derived data compared to published utility values

The literature reviews identified four studies reporting utilities in advanced melanoma. A mapping study by Askew et al. reported a utilities of 0.85 for stage III melanoma (n = 100) and 0.86 for stage IV melanoma (n = 102).[17] Dixon et al., in a trial of interferon-alpha compared to placebo, collected EQ-5D over time, increasing from 0.76 at baseline (n = 111), to 0.77 at 3 months (n = 80), 0.82 at 12 months (n = 66), and finally to 0.83 at 24 months (n = 31) [18]; personal communication with the author. Two vignette studies were also available (bespoke descriptions of health states that are valued by the general public) – one based in Australia/UK (n = 77/63) published by Beusterien et al. [19], and one based in Canada (n = 87). [20] These types of studies are often used in health-state valuation for cost-effectiveness analyses [21-23], especially when patient derived utilities are not available [24]. In the two vignette studies, members of the public were asked to value partial response, giving values of 0.85 and 0.84 in Australia/UK and Canada, stable disease (0.77 and 0.79), progressive disease (0.59 and 0.55), and best supportive care (0.59 and 0.54).

The values seen in MDX010-20 were broadly comparable to the values used in the published literature. Although much higher than utilities typically seen in advanced cancer [25], that values in multiple studies are similar may indicate that patients with advanced melanoma exhibit HRQL similar to age-matched members of the general population [10]. Of particular interest are the values seen in Dixon et al., which showed the quality of life of patients to increase the longer patients were in the study, potentially indicating a link between proximity to death, and low HRQL.

### Progression versus time to death approach

Previous reviews have provided evidence for a positive relationship between HRQL measures and the survival duration of cancer patients [26,27]. A recent review identified three studies in either advanced or metastatic melanoma, all of which show either overall HRQL or certain domains of HRQL to be predictive of survival [28]. The analysis we have performed is therefore consistent with this body of work and attempts to quantify this relationship.

Utilities generated by both the EORTC-8D and SF-6D showed a substantial decrease in utility in the final 180 days before a patient’s death. This was consistent across measures and regression models, and, based on clinician feedback it is possible that this is the case for all metastatic melanoma patients. However, further research is required using different datasets to establish whether this is the case.

Progression status is a standard primary endpoint used in clinical trials to define efficacy, and as such has been the obvious choice for defining health states in cost-effectiveness modelling. Oncology models are frequently based on area under the curve models with three health states (pre-progression, post-progression and death), with transitions driven by parametric curves. However, our research suggests that this approach may not be suitable to model patients’ HRQL if disease progression is not closely related to HRQL. In this case additional health states may need to be added to model the path of HRQL.

If this pattern of HRQL being linked to survival is repeated in other oncology areas, it may be better to replace the current standard method of progression-based utilities, with analysis on a case-by-case basis - including investigation of alternative explanations for HRQL decreases (for example a time to event based approach). Designers of oncology studies should carefully consider the time points at which HRQL is measured, in order to ensure accurate estimates of HRQL can be made both pre- and post-progression. Investigators should also examine utility datasets with a clinical rationale for analyses, and not purely through an economic lens. Whilst it may be that the progression-based model remains a good fit for many cancers, this should be shown with data rather than assumed to be the case.

It is important to note that the effect on the results of economic modelling when using a time to death approach (rather than progression-based approach) for HRQL in a model may be dependent upon the length of time patients live in a post-progression state. Within the MDX010-20 dataset, 17% of ipilimumab treated patients were alive at the end of the 56-month trial period - nearly all of these in a post-progression state [5]. The use of a lower utility than these patients experienced (as would occur when taking the mean utility measurement for progressive disease) over such a substantial time period will substantially worsen the modelled cost effectiveness of a drug. A time-to-death utility may assign a lower utility to patients only shortly before death, thereby increasing the number of modelled quality-adjusted life years, and potentially altering investment decisions made as a result. It should be noted, however, that the cost-effectiveness of treatments would not always improve, and the results seen would depend on individual analyses – a treatment with worse post-progression survival may become less cost-effective.

It is possible that the results seen in MDX010-20 would not be reproduced in other datasets. The MDX010-20 clinical trial was extremely mature, with approximately 90% of enrolled patients having died on completion at 56 months (giving many data points for analysis). Equally the underlying disease of melanoma may result in a different quality of life profile to solid tumours or haematological malignancies, which have been more widely studied. It is also plausible that the results seen were impacted by the treatments used in the MDX010-20 study. In MDX010-20 the control arm was relatively begin (a vaccine with few side effects, later shown to be ineffective). The study drug, ipilimumab, has a defined course of 4 infusions (not on-going treatment, with corresponding adverse events), and a relatively good safety profile. Studies with more toxic chemotherapies including frequent administrations (and more aggressive control arms) may show different results.

Finally, the use of ipilimumab may affect pre- and post-progression utility through a delayed response due to the drug’s mechanism of action – as immunotherapy ipilimumab acts indirectly on cancer cells via the immune system. This means that patients may progress before showing a response at which point their symptoms (and hence HRQL) improve, owing to the delayed reaction of treatment [27]. Although the control arm (gp100) did not show a different pattern to ipilimumab treatment, this may be due to sample size; four-fifths of the patients received ipilimumab, either as monotherapy or in combination with gp100. This delayed reaction therefore may also be sufficient to cause the relatively high quality of life on disease progression.

## Conclusions

This study adds to the body of evidence that suggests alternative quality of life measures produce different results. This can therefore be seen as a validation of the position of the Scottish Medicines Consortium and NICE in requesting a consistent source of utility values [12,29]. The results seen in this study also indicate further research is needed on the relationship between the EORTC-8D, SF-6D and EQ-5D.

In addition, this study demonstrated that utilities based on time to death appear to provide a good fit to patient data in the MDX010-20, when compared to utilities based on disease progression. Practitioners should carefully analyse HRQL data prior to constructing economic models based on clinical trials; clinical measures such as disease progression may not explain quality of life changes, and an event-based approach may be more suitable.

## Additional file

Additional file 1: Table S1. Results of regressions showing the impact of adding melanoma stage, prior interleukin-2, age, and treatment assignment.

## Abbreviations

EORTC: European Organisation for Research and Treatment of Cancer
EQ-5D: EuroQoL 5 dimension
HRQL: Health-related quality of life
MAE: Mean Absolute error
NICE: National Institute for Health and Care Excellence
RMSE: Root mean squared error
