# Supplementary material for: Patient-reported utilities in advanced or metastatic melanoma, including analysis of utilities by time to death
Source: Health Qual Life Outcomes. 2014 Sep 10;12:140. doi: 10.1186/s12955-014-0140-1 (PMC4173059; doi:10.1186/s12955-014-0140-1)
Supplement: Additional file 1: Table S1. — Results of regressions showing the impact of adding melanoma stage, prior interleukin-2, age, and treatment assignment. [file 12955_2014_140_MOESM1_ESM.doc]

Additional Table 1: Results of regressions showing the impact of adding melanoma stage, prior interleukin-2, age, and treatment assignment

| Variable | EORTC-8D | | | SF-6D | | |
| --- | --- | --- | --- | --- | --- | --- |
|  | Coefficient | Standard Error | p-value | Coefficient | Standard Error | p-value |
| Age (years) | 0.000 | 0.000 | 0.501 | 0.000 | 0.000 | 0.869 |
| Prior Interleukin-2 (dummy variable) | 0.000 | 0.012 | 0.976 | 0.015 | 0.010 | 0.149 |
| Melanoma stage M1B (dummy variable) | 0.012 | 0.018 | 0.496 | 0.008 | 0.016 | 0.642 |
| Melanoma stage M1C (dummy variable) | -0.001 | 0.016 | 0.946 | -0.003 | 0.014 | 0.829 |
| Ipilimumab treatment (dummy variable) | 0.009 | 0.012 | 0.446 | 0.016 | 0.011 | 0.139 |
| 120 - 179 days to death (dummy variable) | -0.058 | 0.012 | 0.000 | -0.048 | 0.010 | 0.000 |
| 90 - 119 days to death (dummy variable) | -0.063 | 0.015 | 0.000 | -0.055 | 0.013 | 0.000 |
| 60 - 89 days to death (dummy variable) | -0.104 | 0.016 | 0.000 | -0.083 | 0.014 | 0.000 |
| 30 - 59 days to death (dummy variable) | -0.139 | 0.015 | 0.000 | -0.110 | 0.014 | 0.000 |
| Under 30 days to death (dummy variable) | -0.163 | 0.020 | 0.000 | -0.124 | 0.017 | 0.000 |
| Progressed (dummy variable) | -0.030 | 0.007 | 0.000 | -0.017 | 0.006 | 0.004 |
| Constant | 0.813 | 0.029 | 0.000 | 0.650 | 0.026 | 0.000 |
| The base regression would be a patient of age 0, non-progressed disease, no prior interleukin-2, with stage m1a melanoma (A-C scale, C being more advanced), treated with gp100, more than 180 days from death. | | | | | | |
